# Future carbon emissions from global mangrove forest loss

**DOI:** 10.1111/gcb.15571

**Published:** 2021-03-17

**Authors:** Maria F. Adame, Rod M. Connolly, Mischa P. Turschwell, Catherine E. Lovelock, Temilola Fatoyinbo, David Lagomasino, Liza A. Goldberg, Jordan Holdorf, Daniel A. Friess, Sigit D. Sasmito, Jonathan Sanderman, Michael Sievers, Christina Buelow, J. Boone Kauffman, Dale Bryan‐Brown, Christopher J. Brown

**Affiliations:** ^1^ Australian Rivers Institute Griffith University Nathan Qld Australia; ^2^ Coastal and Marine Research Centre, Australian Rivers Institute, School of Environment and Science Griffith University Gold Coast Qld Australia; ^3^ School of Biological Sciences, The University of Queensland St Lucia Qld Australia; ^4^ NASA Goddard Space Flight Center Greenbelt MD USA; ^5^ Department of Coastal Studies East Carolina University Wanchese NC USA; ^6^ Earth System Science Interdisciplinary Center University of Maryland College Park MD USA; ^7^ Department of Geography National University of Singapore Singapore Singapore; ^8^ Mangrove Specialist Group Centre for Nature‐based Climate Solutions, National University of Singapore Singapore Singapore; ^9^ Research Institute for Environment and Livelihoods Charles Darwin University Casuarina NT Australia; ^10^ Center for International Forestry Research Bogor Indonesia; ^11^ NUS Environmental Research Institute National University of Singapore Singapore Singapore; ^12^ Woodwell Climate Research Center Falmouth MA USA; ^13^ Department of Fisheries, Wildlife and Conservation Sciences Oregon State University Corvallis OR USA

**Keywords:** blue carbon, carbon sequestration, climate change, coastal wetlands, erosion, greenhouse gases, Nationally Determined Contributions, tropical storms

## Abstract

Mangroves have among the highest carbon densities of any tropical forest. These ‘blue carbon’ ecosystems can store large amounts of carbon for long periods, and their protection reduces greenhouse gas emissions and supports climate change mitigation. Incorporating mangroves into Nationally Determined Contributions to the Paris Agreement and their valuation on carbon markets requires predicting how the management of different land‐uses can prevent future greenhouse gas emissions and increase CO_2_ sequestration. We integrated comprehensive global datasets for carbon stocks, mangrove distribution, deforestation rates, and land‐use change drivers into a predictive model of mangrove carbon emissions. We project emissions and foregone soil carbon sequestration potential under ‘business as usual’ rates of mangrove loss. Emissions from mangrove loss could reach 2391 Tg CO_2 eq_ by the end of the century, or 3392 Tg CO_2 eq_ when considering foregone soil carbon sequestration. The highest emissions were predicted in southeast and south Asia (West Coral Triangle, Sunda Shelf, and the Bay of Bengal) due to conversion to aquaculture or agriculture, followed by the Caribbean (Tropical Northwest Atlantic) due to clearing and erosion, and the Andaman coast (West Myanmar) and north Brazil due to erosion. Together, these six regions accounted for 90% of the total potential CO_2 eq_ future emissions. Mangrove loss has been slowing, and global emissions could be more than halved if reduced loss rates remain in the future. Notably, the location of global emission hotspots was consistent with every dataset used to calculate deforestation rates or with alternative assumptions about carbon storage and emissions. Our results indicate the regions in need of policy actions to address emissions arising from mangrove loss and the drivers that could be managed to prevent them.

## Graphical abstract

Business as usual for mangroves by the end of the century could result in emissions of over 2300 Tg of CO_2_ from agriculture in southeast and south Asia, and from clearing and erosion in the Caribbean, Myanmar, and north Brazil.

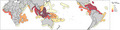

## 1 INTRODUCTION

The capacity of mangroves to store carbon and mitigate greenhouse gas emissions became prominent a decade ago (Donato et al., 2011). Since then, mangroves have gained international interest for their potential to contribute to carbon mitigation strategies and for their ecosystem services that support adaptation to climate change (Lovelock & Duarte, 2019). Hundreds of site‐scale studies have been conducted to understand the distribution and accumulation of mangrove soil carbon and aboveground biomass (Kauffman et al., 2020). These site‐scale measurements have supported globally comprehensive spatial models of carbon storage (e.g. Rovai et al., 2018; Sanderman et al., 2018; Simard et al., 2019). Simultaneously, global efforts to accurately map and monitor mangrove cover and health have provided unprecedented knowledge on the risks that mangrove forests face (Bunting et al., 2018; Goldberg et al., 2020; Hamilton & Casey, 2016). These studies have enabled global‐scale estimation of mangrove carbon storage and its historical loss across different nations (Murdiyarso et al., 2015; Serrano et al., 2019) and globally (Atwood et al., 2017).

Management actions, such as avoiding deforestation or restoring hydrological connectivity, can reduce CO_2_ emissions from mangrove loss and enhance the sequestration potential of disturbed forests (Friess, Krauss, et al., 2020; O’Connor et al., 2020). But management actions should be guided by predictions of future emissions, not just carbon storage. Management effectiveness relies on understanding the level of emissions that can be avoided by specific actions, for instance, by reducing land conversion or increasing restoration efforts. Predictions of CO_2_ emissions from mangrove loss linked with specific land‐use changes can underpin the selection of actions to support adequate mangrove management actions for specific loss drivers. These actions include improving mangrove representation in the Nationally Determined Contributions committed in the Paris Climate Agreement, strengthening their role as natural‐based solutions, and improving their valuation in carbon markets (Adame et al., 2018; Seddon et al., 2019).

Recent advances in mapping mangrove areas, rates of loss, carbon storage, and emission factors now enable predictions of CO_2_ emissions at the global scale (Worthington et al., 2020). These predictions should overcome several critical limitations of past studies (Macreadie et al., 2019). First, estimates have yet to associate particular land‐use changes with CO_2_ emissions, as global mapping of mangrove loss drivers has just recently become available (Goldberg et al., 2020). Second, many global estimates have included only the first metre of soil, thus underestimating the total carbon content and the emissions that arise from mangrove conversion to other land‐uses (Kauffman et al., 2020). Third, estimates of global carbon emissions have not included the foregone carbon sequestration, and they do not account for the lost opportunity of sequestration when mangroves are lost (Maxwell et al., 2019). And finally, global estimates have treated all CO_2_ emissions from mangroves as occurring in the year of loss (Atwood et al., 2017). Depending on the type of land‐use change and the carbon pool affected, it can take years or even decades for the carbon stored in mangroves to be emitted into the atmosphere (Lovelock, Fourqurean, et al., 2017) and exported through tidal exchange (Maher et al., 2013).

To overcome current limitations in global estimations, we developed a spatial model that projects emissions caused by mangrove loss. Our model synthesised information from multiple newly available global datasets, including carbon stocks (Kauffman et al., 2020; Sanderman et al., 2018; Simard et al., 2019), mangrove distribution (Bunting et al., 2018), deforestation rates (Goldberg et al. 2020; Hamilton & Casey, 2016), drivers of land‐use change (Goldberg et al., 2020) and emission factors (Sasmito et al., 2019). We provide predictions of future CO_2_ emissions from mangrove loss, accounting for the effect of proximate drivers of land‐use change including: (a) conversion to commodities, such as agriculture or aquaculture; (b) coastal erosion; (c) clearing; (d) extreme climatic events; and (e) conversion to human settlements (Goldberg et al., 2020). Importantly, we account for the foregone opportunity of soil carbon sequestration when mangroves are lost (Maxwell et al., 2019). Our modelled emissions reflect the realistic temporal scale of emissions: not annual, but decadal (Lovelock, Feller, et al., 2017; Lovelock, Fourqurean, et al., 2017). Although predictions may vary due to possible changes in future deforestation rates, we provide the business as usual (BAU) scenario to assess current management practices. For instance, the Global Mangrove Alliance's commitment to restoring 20% of mangrove cover and reduce emissions from their loss (http://www.mangrovealliance.org/initiatives/). By linking emissions with specific drivers of land‐use change and accounting for future removals, we provide for the first time, spatially explicit information on how different drivers of mangrove loss are causing CO_2_ emissions. We identify and discuss potential options to reduce these emissions and the management actions that could prevent them.

## 2 METHODS

### 2.1 Mangrove area, rates of loss and drivers of change

**FIGURE 1:**
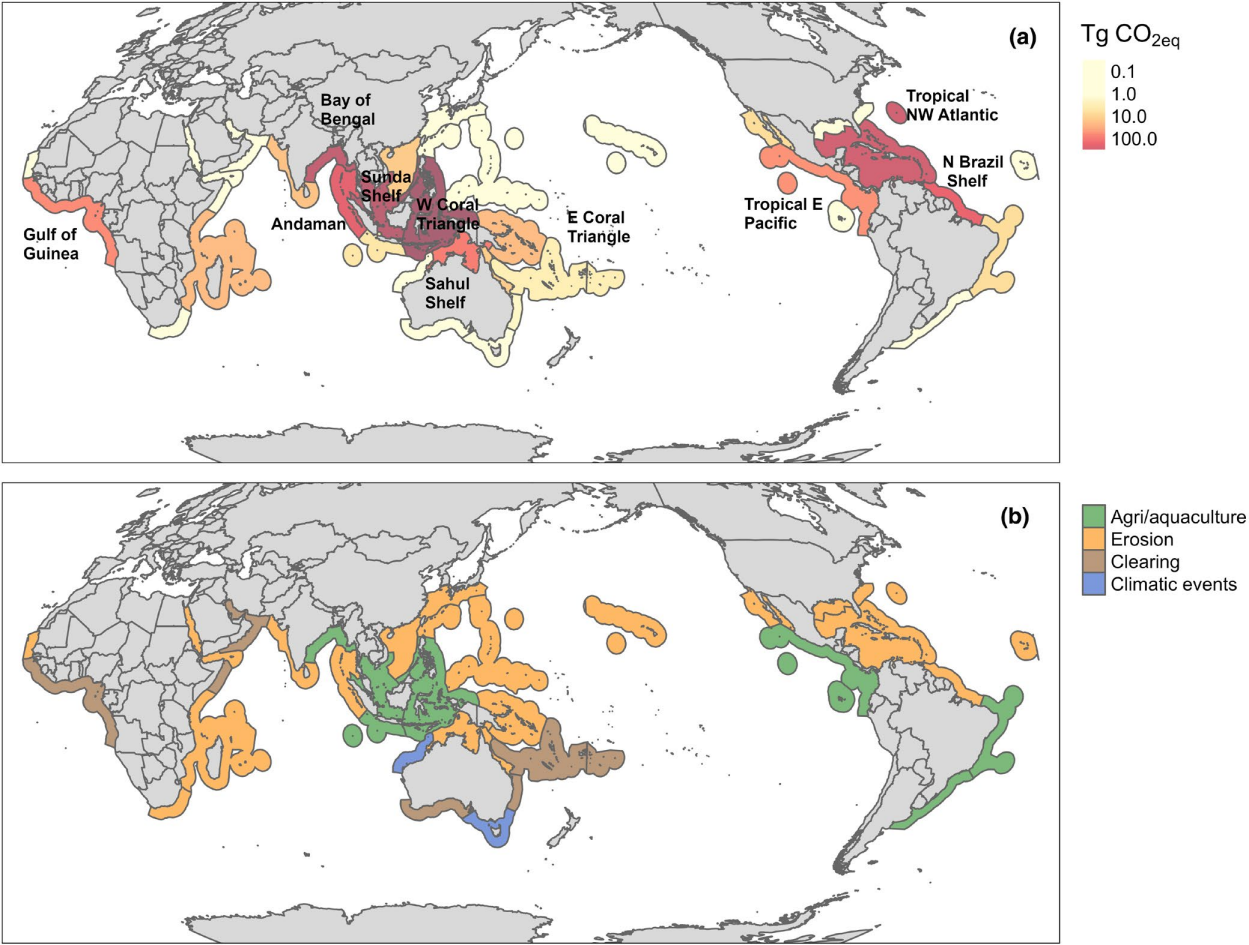
(a) Global projected CO_2 eq_ emissions (Tg) by the end of the century (2010–2100) for the marine provinces of the world and (b) the proximate driver responsible for the largest CO_2_ emissions for each marine province (Goldberg et al., 2020). The names and location for all marine provinces can be found in Figure S1

We divided the global mangrove extent of 2010 (Bunting et al., 2018) into marine provinces (top‐level category of the bioregions) that contained mangroves (Spalding et al., 2007; Van der Stocken et al., 2019; Figure 1; Figure S1; Table S1). We selected this approach to estimate global CO_2_ emissions because it is well aligned with climatic and geomorphic characteristics of mangroves, which are variables associated with carbon stocks and losses (Dürr et al., 2011; Rogers et al., 2019). Deforestation rates for each province were obtained from the dataset by Hamilton and Casey (2016) for 2000–2012. We selected these two datasets (Bunting et al., 2018; Hamilton & Casey, 2016) as they are currently the most accurate datasets to estimate mangrove area and deforestation rate. However, due to the differences in temporal and spatial resolutions, they should be considered general trends within each marine province. To determine how sensitive our future predictions were to each of the variables selected, we conducted sensitivity analyses to repeat the predictions with different datasets for mangrove area (Bunting et al., 2018; Hamilton & Casey, 2016) and deforestation rates (Goldberg et al. 2020: 2010–2016 vs. Hamilton & Casey, 2016: 2010–2012).

The drivers of mangrove loss for each province (2000–2016) were obtained from changes in mangrove area and a decision‐tree model that separated the causes of loss into five categories: (a) conversion to commodities, such as agriculture or aquaculture; (b) coastal erosion; (c) clearing due to various activities including logging or hydrological modifications; (d) extreme climatic events, such as tropical storms and fluctuations in sea level; and (e) conversion to human settlements (Goldberg et al., 2020). Briefly, mangrove loss was estimated from the Surface Reflectance Tier‐1 Landsat 5 TM, 7ETM+ and 8OLI imagery within Google Earth Engine. A baseline period (1999–2001) of a normalised difference vegetation index (NDVI) optimised mosaic representing the year 2000 was created from where mangrove area change was estimated. A threshold change value of −0.2 that occurred within the Mangrove Forests of the World extent (Giri et al., 2011) was used to indicate the areas of mangroves that had transitioned from forest to no‐forest (Lagomasino et al., 2019). A random forest classification was applied to the areas showing a drop in NDVI greater than or equal to 0.2. These areas were trained for each land cover type: water, dark soils and bright soils. Erosion was defined as a transition to water that intersected rivers and coastlines. Commodities (agriculture/aquaculture) were defined where mangrove loss intersected the Global Food Security‐support Analysis Data Cropland Extent 30‐m (GFSAD‐30) layer (www.usgs.gov/centers/wgsc/science/global‐food‐security‐support‐analysis‐data‐30‐m). Human settlements were defined as the bright soil land cover class that intersected with the Global Human Settlement Layer (GHSL). Clearing or non‐productive conversions were defined at bright and dark soil land cover intersected with a 5 km buffer around the GRIP‐4 global roads dataset (doi.org/10.7927/H4VD6WCT) and the GHSL dataset (ghsl.jrc.ec.europa.eu/data.php). Finally, conversion by extreme climatic events was defined as other areas where mangroves were lost that did not occur within a 5‐km infrastructure buffer.

### 2.2 Total ecosystem carbon stocks

Total ecosystem carbon stocks (TECS) were obtained from the sum of soil organic carbon (SOC) and aboveground carbon (ABC). Stocks for 1 and 2 m of soil were obtained from the global SOC dataset (Sanderman et al., 2018), derived from a random forest model trained on field measurements. For ABC, biomass was obtained from the global dataset of mangrove biomass (Simard et al., 2019). The total biomass per province was divided by mangrove area to get a mean ABC per province and multiplied by a factor of 0.48 to obtain carbon values (Kauffman & Donato, 2012). To test the accuracy of the global‐scale model, we compared the TECS obtained from the global models with verified field measurements from provinces where data were available (Kauffman et al., 2020) with a linear regression (IBM SPSS Statistics, v25). TECS obtained from global models (Sanderman et al., 2018; Simard et al., 2019) were lower in provinces with high stocks (>1200 MgC ha^−1^, e.g. Sunda Shelf and West Coral Triangle) and higher in provinces with small stocks (<220 MgC ha^−1^, e.g. Northwest Australian Shelf and Somali Arabian). The predicted values from the global model were close to the field measurements when including SOC for 2 m in depth (Figure S2; Table S1). Hence, we calculated TECS for all provinces as the sum of ABC and SOC for the top 2 m of soil.

### 2.3 Emission factors

The emission factor is the fraction of carbon that is emitted given conversion to a specific land‐use change. We selected an emission factor for each province and activity from a recent global systematic review (Sasmito et al., 2019). Each emission factor was given a level of confidence (Table S2) from low to high, with Level 1 (lowest confidence) given to emission factors obtained from a global average; Level 2 to those obtained from a similar region; and Level 3 (highest confidence), from a similar region with the same geomorphic setting (following Dürr et al., 2011).

### 2.4 Model for projecting emissions and missed opportunities to sequester carbon

We updated a model of carbon emissions from deforested mangroves (Adame et al., 2018) to account for drivers of land‐use change and SOC sequestration. The model allowed for variable carbon stocks across discrete spatial units and assumed a constant rate of deforestation and a constant rate of emissions once mangroves were lost. We modelled foregone carbon sequestration from mangrove loss in each province as the difference between carbon storage with deforestation and a counterfactual with no deforestation:

(1) $$L_{t}=C_{t}^{0}‐C_{t}^{d}.$$

Cumulative carbon emissions, *L_t_*, were described by three dynamic equations:

(2) $$\frac{dA}{dt}=‐Ad,$$

(3) $$\frac{dE}{dy}=A_{1}\cdot d\cdot e^{‐dt}\cdot c\cdot r\cdot e^{‐\left(t‐y\right)r},$$

(4) $$\frac{dS}{dt}=s\cdot A_{1}e^{‐dt},$$

where *A* is the area of mangroves in hectares, *d* is the total deforestation rate across all land‐uses, *E* is the emissions, *r* is the rate of emissions from deforested mangroves, *c* is the total carbon stock emitted per hectare, *y* is the year of deforestation, *S* is sequestered carbon and *s* is the yearly sequestration rate per hectare.

We assumed that future rates of deforestation due to each of the five drivers were in proportion to their historical contributions to loss from Goldberg et al. (2020). Therefore, province‐specific potential emissions per hectare were scaled by land‐use types and their respective emission factors:

(5) $$c_{j}=c_{j}^{\max}\sum_{i=1}^{5}f_{i,j}p_{i,j},$$

where $c_{j}^{\text{max}}$ is maximum labile carbon per hectare for a province including SOC and AGC, $f_{i,j}$ are province and land‐use specific emission factors and $p_{i,j}$ are the proportional contributions of each land‐use type to past deforestation.

### 2.5 Management recommendations

Based on the emissions predicted for each province and the specific drivers for mangrove loss, we discuss possible management actions. For instance, if a province has over 80% of emissions due to conversion to agriculture or aquaculture, we discuss, in the context of the region, management actions that could improve these activities and reduce emissions.

### 2.6 Sensitivity analyses to global datasets and model robustness

To determine how sensitive our future predictions were to each of the variables selected, we conducted sensitivity analyses. We ran the model with different datasets of mangrove area (Bunting et al., 2018; Hamilton & Casey, 2016), sources of data (modelled and field; Kauffman et al., 2020; Sanderman et al., 2018; Simard et al., 2019), SOC depth (1 m, 2 m and whole sediment column) and deforestation rates (Goldberg et al., 2020; Hamilton & Casey, 2016). Recent analyses suggest that rates of mangrove deforestation may be slowing (Friess, Yando, et al., 2020; Goldberg et al., 2020). Therefore, we compared our base scenario to predictions that used deforestation rates from 2010 to 2016 in Goldberg et al. (2020). We also conducted sensitivity analyses on the emission factors relating to erosion and extreme climatic events, which can be highly variable (Sasmito et al., 2019). Erosion can cause large emissions in one location, but these can be partly compensated by mangrove accretion in other location (Lagomasino et al., 2019). Extreme climatic events, such as tropical storms, can cause large‐scale mortality; however, some areas can naturally recover after a few years if conditions are appropriate, thereby reducing emissions (Krauss & Osland, 2020). We implemented the model with emission factors 50% and 100% for erosion, and with and without mangrove area loss from climatic events. Finally, we conducted further formal sensitivity analyses of the model to all the parameter inputs by taking the derivative of *L_t_* (cumulative carbon emissions) with respect to each parameter.

## 3 RESULTS

### 3.1 Inputs to the model

First, we present summaries of the input data, noting that these data have been reported elsewhere, but not aggregated by provinces. The mean TECS (mean ± SE, [range]) measured in the field for all provinces was 624.5 ± 96.9 (181.5–1434.9) Mg C ha^−1^. The mean modelled SOC in the top metre of soil was 331.3 ± 74.9 (207.4–497.8) Mg C ha^−1^, in the top 2 m was 646.7 ± 150.6 (408.6–975.9) Mg C ha^−1^ and mean ABC was 101.2 ± 93.5 (9.9–466.0) Mg C ha^−1^. Ten provinces contained 88% of all the mangroves in the world, with largest areas of mangroves in the West Coral Triangle, the Gulf of Guinea, Sahul Shelf and Tropical Northwest Atlantic (Table S1; Figure S3). From 2000 to 2012, 35 of the 37 provinces had some level of deforestation, with mean annual losses of 0.09 ± 0.02%. The highest deforestation rates were in the Bay of Bengal (0.55%), Sunda Shelf (0.35%), West Coral Triangle (0.33%) and Tropical Northwest Atlantic (0.14%) (Table S1; Figure S3). In general, the area of mangrove lost between 2000 and 2012 was proportional to total mangrove area (Table S1). However, there were some exceptions; for instance, the Bay of Bengal was seventh in mangrove area (911,223 ha), but had the second largest mangrove loss; comparatively, the Gulf of Guinea was second in mangrove area (1,806,989 ha), but was seventh in mangrove loss.

Conversion of mangroves to aquaculture/agriculture was the primary proximate driver of mangrove loss, which caused the conversion of 219,392 ha of mangroves from 2000 to 2016, especially in the West Coral Triangle, Bay of Bengal and Sunda Shelf (Figure 1a; Table 1). This corresponds to 87%, 74%, and 70% of their total mangrove loss respectively. The second most important proximate driver of mangrove loss was erosion, which caused the loss of 92,787 ha, mainly in North Brazil Shelf (55% of the total mangrove loss of the province), the Bay of Bengal (19%) and Sunda Shelf (19%). The third proximate driver of mangrove loss was extreme climatic events, causing the loss of 41,525 ha of mangroves, mainly in Sahul Shelf (42%), Tropical Northwest Atlantic (31%) and North Brazil Shelf (6%). The fourth most important driver was mangrove clearing which caused the loss of 39,595 ha, mostly in the Gulf of Guinea (42%), West Indian Ocean (36%) and Tropical Northwest Atlantic (31%). Finally, the fifth proximate driver of mangrove loss was human settlement, which caused the loss of 10,529 ha, mostly in the Gulf of Guinea (16%) and Sunda Shelf (6%).

**TABLE 1:** Annual mangrove loss (ha year^−1^; 2000–2016) and cumulative emissions (Tg CO_2 eq_) projected for the next century (2010–2100) derived by agri/aquaculture, erosion, clearing, extreme climatic events and human settlements for the top six emitting marine provinces of the world. For the full list of provinces, see Table S3

Marine province	Agri/aquaculture		Erosion		Clearing		Extreme climatic events		Human settlements	
	ha	Tg CO_2 eq_	ha	Tg CO_2 eq_	ha	Tg CO_2 eq_	ha	Tg CO_2 eq_	ha	Tg CO_2 eq_
West Coral Triangle	6,264	519.9	681	163.8	152	18.2	43	1.7	50	8.5
Sunda Shelf	2,783	221.3	741	173.4	128	14.7	49	1.8	249	40.6
Bay of Bengal	3,413	243.7	894	112.2	171	8.4	150	3.1	14	1.2
Tropical Northwest Atlantic	66	9.1	672	190.8	578	79.9	516	22.7	49	9.6
Andaman	168	41.9	130	97.5	39	14.4	54	6.4	2	1.2
North Brazil Shelf	383	21.9	1,284	103.5	132	4.1	538	7	2	0.1

### 3.2 Predictions of carbon emissions and lost opportunities to sequester carbon

**FIGURE 2:**
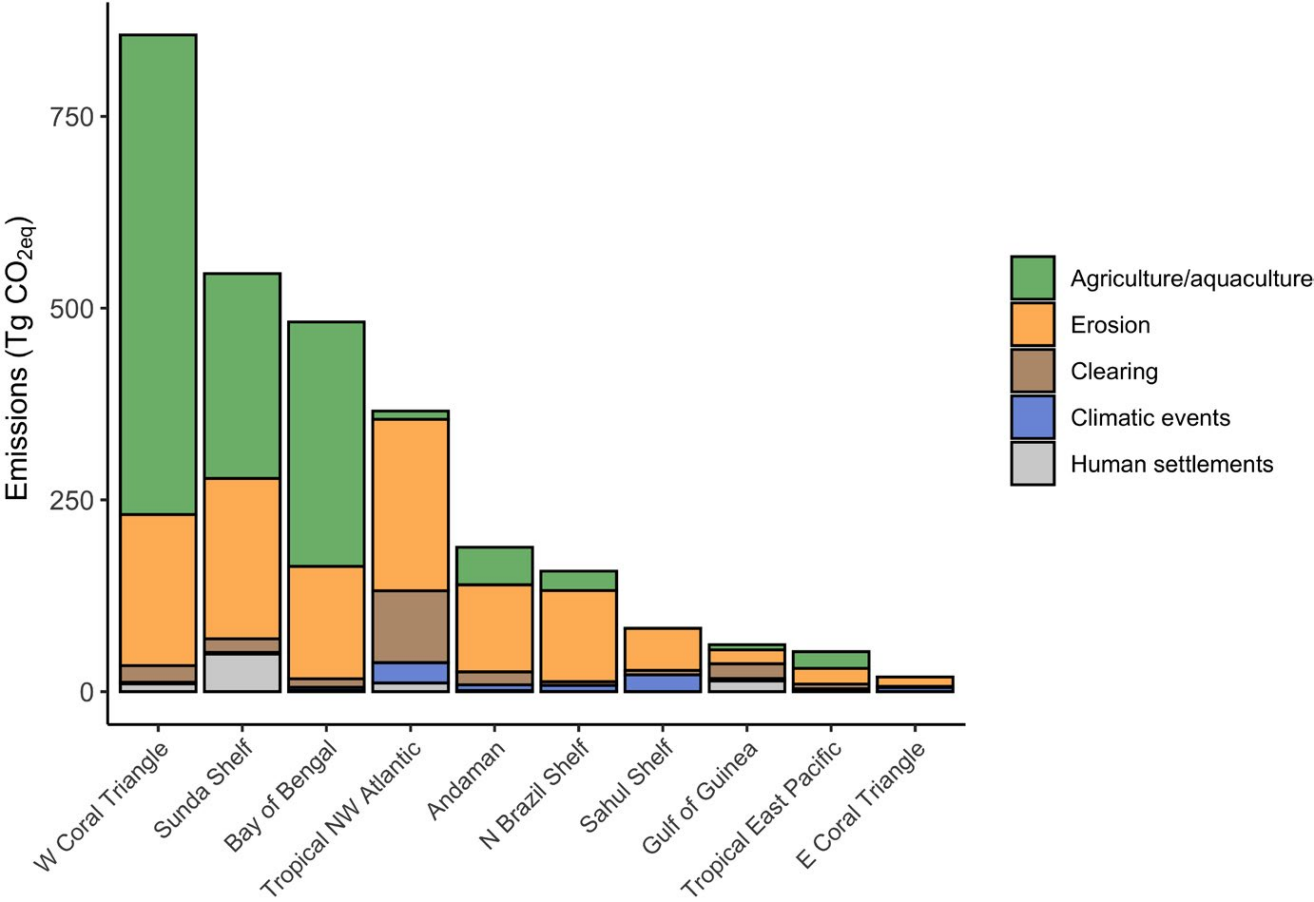
Cumulative CO_2 eq_ emissions (Tg) by the end of the century (2010–2100) attributed to the proximate drivers of mangrove loss for the marine provinces ranked in the top ten for future CO_2_ emissions

Global emissions from mangrove loss are projected to reach 2391 Tg CO_2 eq_ by the end of the century (2020–2100). Including the loss of potential carbon sequestration once mangroves are deforested (considered to have a global mean value of 1.5 Mg C ha^−1 ^year^−1^; Alongi, 2014) increased our projection to 3392 Tg CO_2 eq_. Projected CO_2_ emissions showed significant geographical variability (Figure 1a). They were highest for the West Coral Triangle (712 Tg CO_2 eq_), followed by Sunda Shelf (452 Tg CO_2 eq_), Bay of Bengal (369 Tg CO_2 eq_), Tropical Northwest Atlantic (312 Tg CO_2eq_), Andaman coast (161 Tg CO_2 eq_) and North Brazil Shelf (137 Tg CO_2 eq_). Collectively, these six provinces contributed 90% of the total projected global CO_2_ emissions (Figures 1a and 2; Table 1).

The West Coral Triangle, Sunda Shelf and the Bay of Bengal had the highest predicted emissions due to mangrove conversion to agriculture/aquaculture at 985 Tg CO_2 eq_, contributing 73% to its emissions (Figures 1b and 2; Table 1). Additionally, erosion was an important driver of mangrove loss in these provinces, accounting for 23%, 38% and 30% of their emissions respectively (Figure 2). Similarly, the adjacent province of Andaman (west Myanmar, Bangladesh and East India) had significant emissions due to erosion (98 Tg CO_2 eq_ or 60% of its total emissions). A second hotspot for mangrove CO_2_ emissions was identified in the Tropical Northwest Atlantic, which had large emissions due to erosion (191 Tg CO_2 eq_), clearing (80 Tg CO_2 eq_) and extreme climatic events (23 Tg CO_2 eq_), with total emissions projected to reach 312 Tg CO_2 eq_ by the end of the century (Figures 1 and 2; Table 1). Smaller hotspots with lower CO_2_ emissions were predicted to occur on the North Brazil Shelf, Sahul Shelf, Gulf of Guinea, Tropical East Pacific and East Coral Triangle (Figures 1a and 2).

## 4 DISCUSSION

Under a BAU scenario, global emissions from mangrove loss could reach 2,391 Tg CO_2 eq_ by the end of the century (2010–2100), or 3392 Tg CO_2 eq_ if considering the lost opportunity for soil carbon sequestration. Previous estimates of mangrove emissions for the same period vary enormously, between 630 and 40,230 Tg CO_2 eq_ (Friess, Krauss, et al., 2020)_._ Our projection lies towards the lower end of this range, and we consider it more accurate because of the inclusion of land‐use drivers, time lags and foregone future sequestration that were not considered in previous studies.

**FIGURE 3:**
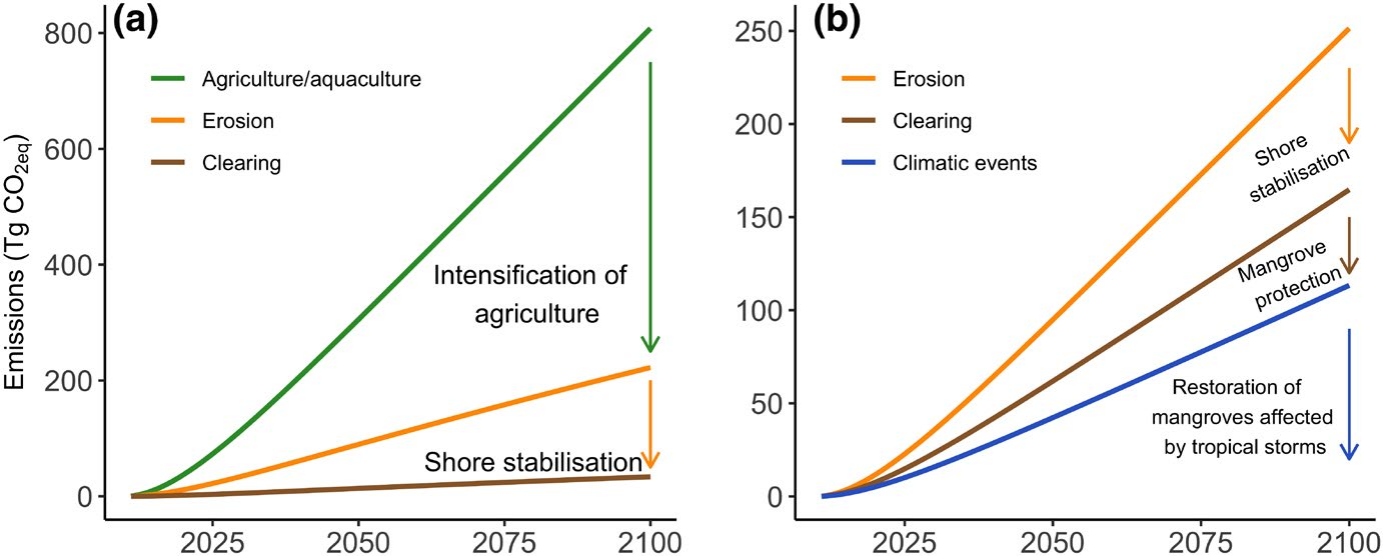
Emission reductions (Tg CO_2 eq_) that could be achieved from (a) management of agriculture/aquaculture and shore stabilisation in the West Coral Triangle and (b) decrease in erosion through shore stabilisation, mangrove protection to avoid clearing and restoration of mangroves affected by tropical storms in the Tropical Northwest Atlantic

We identified six provinces that accounted for 90% of the projected emissions. The top emitters were the West Coral Triangle, Sunda Shelf and the Bay of Bengal, primarily due to agriculture/aquaculture conversion. These regions have been previously highlighted as a global hotspot of mangrove CO_2_ emissions (Atwood et al., 2017). Within these provinces, clearing of large areas of carbon‐rich mangroves has occurred for rice, oil palm, aquaculture and rubber plantations (De Alban et al., 2019; Richards & Friess, 2016). In Indonesia, the conversion of mangroves to aquaculture contributed almost 15% of their national emissions (Murdiyarso et al., 2015). In Myanmar, deforestation of mangroves has been driven by national policies that support the intensification of rice production to increase food security (Webb et al., 2014). Our predictions suggest that emissions from these regions will be the highest globally by the end of the century due to the intensity of land‐use changes and large mangrove carbon stocks. These emissions can be managed through changes in agricultural practices, and the restoration of formerly converted mangrove areas, such as disused aquaculture ponds and on land where saltwater has intruded (Figure 3a).

Erosion was an important driver of mangrove loss and was responsible for significant carbon emissions, particularly within the West Coral Triangle, Sunda Shelf, Bay of Bengal and Andaman provinces. In the Sundarbans, changes in river flows have reduced sediment inputs, which caused the loss of over 7500 ha of coastline in the last 37 years (Bhargava et al., 2020). In areas prone to high erosion rates, decreasing emissions would need to be achieved through shore stabilisation and the management of rivers and dams to provide sediment inputs that support the maintenance of surface elevation and habitat area for mangroves (Lovelock et al., 2015; Figure 3a). Landward migration of mangroves, if coastal squeeze is avoided, may also balance some losses in provinces with high levels of erosion (Schuerch et al., 2018).

The second global hotspot for mangrove CO_2_ emissions was the Tropical Northwest Atlantic province, driven primarily by erosion, clearing and extreme climatic events. In the Mexican Caribbean, changes in hydrological connectivity that affect groundwater are a significant cause of unintended clearing of mangroves rich in carbon (Adame et al., 2013). The Tropical Northwest Atlantic is also one of the regions with the highest frequency of tropical storms in the world, which can cause large‐scale mangrove mortality (Krauss & Osland, 2020). Management activities to decrease CO_2_ emissions in the Tropical Northwest Atlantic could include coastal stabilisation, reduction of illegal deforestation and improvement of hydrological connectivity, especially in sites that fail to recover after tropical storms (Zaldívar‐Jiménez et al., 2010). These activities combined could reduce the projected carbon emissions by 94% for this region (Figure 3b).

Finally, provinces considered smaller hotspots, with an intermediate mangrove area and moderate carbon stocks, were North Brazil Shelf, Sahul Shelf and Gulf of Guinea. In Brazil, vegetation clearing, changes in hydrology and coastal development have increased erosion, leading to mangrove loss (Krause & Soares, 2004). Across the Sahul Shelf, northern Australia, the loss of mangroves during 2015–2016 was associated with an extreme El Niño event which caused fluctuating sea levels, drought and high temperatures (Lovelock, Feller, et al., 2017). In Senegal, in the Gulf of Guinea, drought and hydrological changes caused the loss of large areas of mangroves, and consequent large CO_2_ emissions (Sakho et al., 2017). In these regions, coastal stabilisation, hydrological reconnection and restoration could help reduce potential future emissions.

### 4.1 Sensitivity of predictions to input data sources and limitations

**FIGURE 4:**
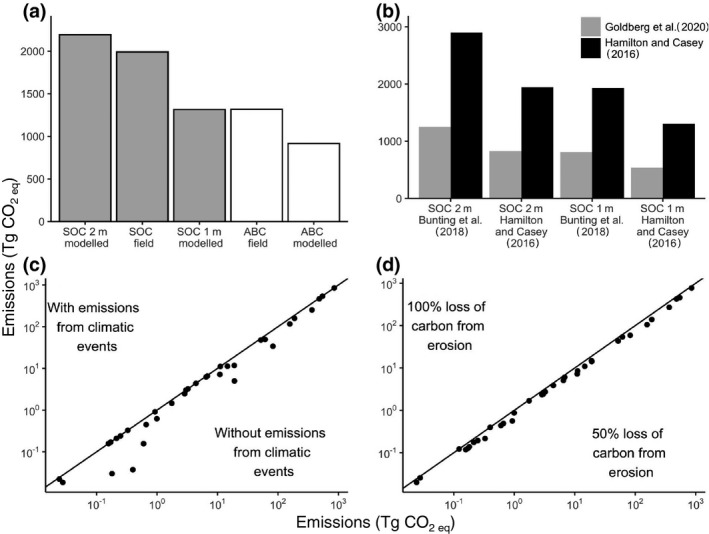
Sensitivity analyses comparing cumulative CO_2 eq_ emissions at the end of the century (2010–2100) using (a) soil organic carbon (SOC) modelled (1 and 2 m deep; Sanderman et al., 2018), SOC obtained in the field (Kauffman et al., 2020) and modelled aboveground biomass carbon (ABC; Simard et al., 2019) and obtained in the field for the 15 provinces with data (Kauffman et al., 2020); (b) comparison between emissions with mangrove area from Bunting et al. (2018) and Hamilton and Casey (2016) with SOC at 1 and 2 m, and with deforestation rates of Goldberg et al. (2020) and Hamilton and Casey (2016); (c) comparison of ranking among provinces with highest cumulative CO_2 eq_ emissions with and without accounting for emissions from climate events, and (d) with low (50%) and high (100%) emission factors from erosion

The sensitivity analysis demonstrated that the spatial distribution of our projected CO_2_ emissions hotspots is robust to different datasets of mangrove area, carbon stocks, emission factors and deforestation rates (Figure 4; Figure S4). The six highest provinces for emissions under the Hamilton and Casey (2016) deforestation rates were within the eight top provinces estimated with the Goldberg et al. (2020) rates. However, the total amount of emissions was affected by the input datasets (Figure 4). Global emissions predictions based on the mangrove distribution dataset of Global Mangrove Watch (Bunting et al., 2018) were higher than those derived from the Hamilton and Casey (2016) dataset. The former is considered a more comprehensive representation of mangrove forests globally because it captures mangroves of short stature. For instance, we found that emissions in provinces where short‐statured mangroves are dominant (e.g. Tropical Northwest Atlantic) almost tripled when using the mangrove area from Global Mangrove Watch. The model was also sensitive to emission rates, but only in the short term (Figures S5–S8).

The sensitivity analysis indicated the model was most sensitive to deforestation rates, with emissions increasing linearly as deforestation rate increased (Figure S7). Total global emissions were much lower when using the deforestation rates of Goldberg et al. (2020) compared to those estimated from Hamilton and Casey (2016; Figure 4b). We assumed that future loss rates due to each of the five drivers were proportional to their historical contributions. Therefore, our predictions may overestimate emissions in regions where mangrove deforestation rates are slowing because of policy changes (Friess, Krauss, et al., 2020, Friess, Yando, et al., 2020; Richards et al., 2020). Changes in the magnitude of drivers of mangrove loss are likely to occur in the future, implying that our assumption of linearity in predictions may not happen. For example, unused agricultural land may transition to mangroves and then to urban settlements. Also, mangrove loss may accelerate because of increased frequency of extreme climatic events, but sea‐level rise could compensate for some of the losses (Schuerch et al., 2018). Future studies should incorporate scenarios where mangrove area increases due to landward or poleward migration as sea‐level rises and winter freezes become less common (Osland et al. 2017). Importantly, a research priority is developing future mangrove loss scenarios that consider not only climatic, but also social and economic drivers of mangrove loss (Duarte et al., 2020).

Changes in mangrove area and drivers of loss also occur at different spatial scales, and in many cases, national or local datasets would be more appropriate to address management issues (Worthington et al., 2020). Ideally, global, national and regional models would be compatible in the future when higher resolution global models of landscape change become available (Worthington et al., 2020). However, our model can be downscaled to account for regional differences in mangrove carbon storage, such as those caused by differences in species composition, forest structure (e.g. in Mexico, Adame et al., 2018) or geomorphological setting (e.g. the Brazilian coast, França et al., 2015). A future research need is to compare local studies to global‐scale studies to assess the accuracy of large‐scale predictions. The models should also be updated with new global datasets as they become available. A strength of our model is that it integrates multiple ‘big’ datasets, which are increasingly being developed to support mangrove conservation (Worthington et al., 2020).

## 5 CONCLUSION

We have identified hotspots of CO_2_ emissions due to mangrove loss associated with various drivers of loss. If these losses continue in the same trajectory, we predict emissions arising from mangrove loss will be concentrated in six provinces of the world: West Coral Triangle, Sunda Shelf, Bay of Bengal, Tropical Northwest Atlantic, Andaman and North Brazil Shelf. These regions have large areas of mangroves (>500,000 ha), relatively high rates of loss (≥0.1% annually) and most have high carbon densities (≥500 Mg C ha^−1^). By accounting for specific mangrove loss drivers and the foregone carbon sequestration potential, we update global estimates and provide specific management actions to minimise future emissions efficiently. For instance, activities that improve agricultural practices to reduce further expansion into mangrove areas and efforts to stabilise coastlines and restore former mangrove areas should be prioritised to decrease emissions from mangrove loss by the end of the century.

## AUTHOR CONTRIBUTIONS

Maria F. Adame, Rod M. Connolly and Christopher J. Brown designed the project with major contributions from Daniel A. Friess and Catherine E. Lovelock; Christopher J. Brown, Mischa P. Turschwell and Jordan Holdorf designed the codes and models; Temilola Fatoyinbo, David Lagomasino and Liza A. Goldberg provided spatial data and analyses of mangrove loss drivers; Jonathan Sanderman provided spatial data and analyses of soil carbon; J. B. Kauffman provided field data; Sigit D. Sasmito and Daniel A. Friess provided data on emission factors; Michael Sievers and Dale Bryan‐Brown conducted spatial analyses. Maria F. Adame wrote the first draft and all authors contributed to editing subsequent drafts.

## Supporting information

Supplementary Material

## Data Availability

Data are available as supplementary information. Correspondence for materials should be addressed to f.adame@griffith.edu.au.
